# The prediction of a pathogenesis-related secretome of *Puccinia helianthi* through high-throughput transcriptome analysis

**DOI:** 10.1186/s12859-017-1577-0

**Published:** 2017-03-11

**Authors:** Lan Jing, Dandan Guo, Wenjie Hu, Xiaofan Niu

**Affiliations:** 1 Department of Plant Pathology, Inner Mongolia Agricultural University, Hohhot, 010019 China; 2 Division of Plant Sciences, University of Missouri, Columbia, MO 65211 USA

**Keywords:** *Puccinia helianthi* Schw., Secretory protein, Signal peptide, Prediction algorithm, Bioinformatics

## Abstract

**Background:**

Many plant pathogen secretory proteins are known to be elicitors or pathogenic factors,which play an important role in the host-pathogen interaction process. Bioinformatics approaches make possible the large scale prediction and analysis of secretory proteins from the *Puccinia helianthi* transcriptome. The internet-based software SignalP v4.1, TargetP v1.01, Big-PI predictor, TMHMM v2.0 and ProtComp v9.0 were utilized to predict the signal peptides and the signal peptide-dependent secreted proteins among the 35,286 ORFs of the *P. helianthi* transcriptome.

**Results:**

908 ORFs (accounting for 2.6% of the total proteins) were identified as putative secretory proteins containing signal peptides. The length of the majority of proteins ranged from 51 to 300 amino acids (aa), while the signal peptides were from 18 to 20 aa long. Signal peptidase I (SpI) cleavage sites were found in 463 of these putative secretory signal peptides. 55 proteins contained the lipoprotein signal peptide recognition site of signal peptidase II (SpII). Out of 908 secretory proteins, 581 (63.8%) have functions related to signal recognition and transduction, metabolism, transport and catabolism. Additionally, 143 putative secretory proteins were categorized into 27 functional groups based on Gene Ontology terms, including 14 groups in biological process, seven in cellular component, and six in molecular function. Gene ontology analysis of the secretory proteins revealed an enrichment of hydrolase activity. Pathway associations were established for 82 (9.0%) secretory proteins. A number of cell wall degrading enzymes and three homologous proteins specific to *Phytophthora sojae* effectors were also identified, which may be involved in the pathogenicity of the sunflower rust pathogen.

**Conclusions:**

This investigation proposes a new approach for identifying elicitors and pathogenic factors. The eventual identification and characterization of 908 extracellularly secreted proteins will advance our understanding of the molecular mechanisms of interactions between sunflower and rust pathogen and will enhance our ability to intervene in disease states.

**Electronic supplementary material:**

The online version of this article (doi:10.1186/s12859-017-1577-0) contains supplementary material, which is available to authorized users.

## Background

Sunflower rust, caused by *Puccinia helianthi* Schw., is a widespread disease of sunflower (*Helianthus annuus* L.) throughout the world and may cause significant yield losses and loss of seed quality. *P. helianthi* is an obligate pathogen and completes its life cycle on sunflower. Although *P. helianthi* is a pathogen of great economic importance, little is known about the molecular mechanisms involved in its pathogenicity and host specificity.

Pathogen secretory proteins and host plant defense interactions involve complex signal exchanges at the plant surface and at the interface between the pathogen and the host [1, 2]. Plant pathogens are endowed with a special ability to interfere with physiological, biochemical, and morphological processes of the host plants through a diverse array of extracellular effectors. These are present or active at the intercellular interface or delivered inside the host cell to reach their cellular target and facilitate infection or trigger defense responses [3–5]. Thus, genes encoding extracellular proteins have a higher probability of being involved in virulence.

Many *Avr* genes encoding secreted proteins were identified from haustoria-forming pathogens, such as *AvrL567*, *AvrM*, *AvrP4*, and *AvrP123* in flax rust caused by *Melampsora lini* [6, 7], *AvrPi-ta* and *AvrPiz-t* in rice blast *Magnaporthe grisea* [8, 9], *Avr1b-1* in stem and root rot of soybean *Phytophthora sojae* [10], *Avr3a* in potato late blight *P. infestans* [11], and *ATR13* and *ATR1* in downy mildew of *Arabidopsis* caused by *Hyaloperonospora parasitica* [12, 13]; all of which exhibit pathogenic functions during pathogen infection. In addition, some cell wall degrading enzymes (CWDEs) produced by pathogens are secretory proteins, such as the wood Xylanase Xyn22 and Xyn33 of *M. grisea* [14], and pectinlyase Pmr6 of *Erysiphe cichoracearum* [15]. Some virulence-related proteins, such as Gas1 and Gas2 (expressed specifically at the appressorium formation stage) [16], hydrophobic protein Mpg1 [17], tetraspanin-like protein Pls1 [18] and chitin binding protein Cbp1 of rice blast [19] are in the same category.

Amino terminal signal peptides are responsible for transporting the virulent factors [20]. The N-terminal signal peptides can be classified into four types based on recognition sequences of signal peptidases. The first class is composed of “typical” signal peptides, which are cleaved by one of the various type I SPases of *Bacillus subtilis* [21–23] and most secretory proteins with this signal peptide are secreted into the extracellular environment. This group also includes signal peptides with a so-called twin-arginine motif (RR-motif) that are transported via the twin-arginine translocation pathway (Tat pathway). In bacteria, the Tat translocase is found in the cytoplasmic membrane and exports proteins to the cell envelope or to the extracellular space [24]. The second class of signal peptides are lipoproteins cleaved by the lipoprotein-specific (type II) SPase of *B. subtilis* (Lsp) [25, 26]. Secretory proteins with the aforementioned signal peptides are transported via the general secretion pathway (Sec-pathway) [27]. The third class constitutes prepilin-like proteins cleaved by the prepilin-specific SPase ComC and the fourth class of signal peptides consists of ribosomally synthesized bacteriocin and pheromone [28, 29]. These signal peptides lack a hydrophobic H-domain and they can be removed from the mature protein by a subunit of the ABC transporter or by specific SPases.

With the development of molecular biology, large scale genome and transcriptome sequencing has been used as an effective method for discovering gene expression profiles and novel genes. Several computer-based prediction algorithms have been used to predict the secretomes from many microbial species, such as *Candida albicans* [30], *P. infestans* [31, 32], *Saccaromyces cerevisiae* [33]*, Agrobacterium tumefaciens* [34], *Fusarium graminearum* [35], *Neurospora crassa* [36], *Verticillium dahliae* [37], *Aspergillus oryzae* [38], *Puccinia striiformis* f. sp*. tritici* [39], and *Colletotrichum graminicola* [40]. These predicted secretomes provide a basis for further investigations using wet-lab procedures or more in-depth computational comparisons of relevant data sets.

An examination of the pathogenesis-related secretome of *P. helianthi* is important for understanding the molecular mechanism of pathogen-host interaction. Here, we generated a high-throughput transcriptome analysis of proteins containing a signal peptide. We analyzed a total of 35,286 ORFs of the *P. helianthi* transcriptome using SignalP v4.1, TMHMM v2.0, TargetP v1.1, TatP v1.0 and big-PI predictor bioinformatics tools to identify secretory proteins.

## Methods

### Isolates and culture conditions

Rust-infected sunflower leaves were collected in paper bags seperately, air dried at room temperature for 24 h and then spores from mature uredial pustules were brushed off the leaves and stored at 4–5 °C. The collected inocula were inoculated on universal susceptible line 7350. After 10–15 days urediospores of a single pustule were used inoculating two weeks old susceptible plants to produce purified isolates. Subsequently, fresh urediniospores of each isolate were collected from rusted leaves by flicking leaves against parchment paper, and then fresh spores were dried for 3 days in a desiccator and stored individually in the refrigerator at 80 °C below zero. In this experiment, the transcriptome data were obtained from *P. helianthi* isolate SY.

### *Puccinia helianthi* transcriptomic data sets

We constructed a *P. helianthi* reference transcriptome for different growing stage urediniospores (0 h fresh urediniospores, 4, and 8 h germinated spores). The cDNA library was sequenced on the Illumina HiSeq™ 2500. For the assembly library, raw reads were filtered to remove those containing an adapter and reads with more than 5% unknown nucleotides. Low quality reads were also removed, in which the percentage of low Q-value (≤10) bases was more than 20%. Clean reads were *de novo* assembled by the Trinity Program yielding 59,409 transcripts with a mean size of 1394 bp. Sequence data has been uploaded to the Short Read Archive (https://www.ncbi.nlm.nih.gov/sra) of the National Center for Biotechnology Information (NCBI); accession number SRP059519. The secretory proteins were predicted according to the N-terminal amino acid sequences of 35,286 ORFs (Additional file 1).

### Prediction and validation of excretory/secretory (ES) proteins

ORFs fulfilling the following four criteria were defined as the computational secretome: (a) the ORF contains an N-terminal signal peptide; (b) the ORF has no transmembrane domains; (c) the ORF has no GPI-anchor site; and (d) the sequence does not contain the localization signal, which may target mitochondria or other intracellular organelles.

Table 1 summarizes the bioinformatic tools used in this study. SignalP v4.1, TMHMM v2.0, TargetP v1.1, ProtComp v9.0 and big-PI predictor tools were employed to identify expected secretory proteins of *P. helianthi*. SignalP predicts classical secretory proteins in eukaryotes and a truncation protein sequence at 70 amino acids as filters. The standard was L = −918.235-123.455* (Mean S score) +1983.44* (HMM score) and L > 0 for predicting signal peptide proteins. TargetP allowed the prediction of mitochondrial proteins with a cut-off of 0.95 for mitochondrial proteins and 0.90 for proteins in other locations. Transmembrane proteins were predicted with TMHMM (version 2.0) with default options. The putative proteins generated from the transcriptome were initially analyzed by SignalP to predict classical secretory proteins on the basis of a D-score greater than 0.5. The proteins identified were then analyzed with TMHMM to screen for classical secretory proteins without transmembrane segments. Proteins that passed the first two steps were then evaluated by TargetP to identify mitochondrial proteins. Once mitochondrial proteins were identified, the remaining secretory proteins were examined and their sub-cellular localization was predicted with Protcomp. Those assigned to extracellular (secreted) categories were considered pathogenic secretory proteins.

**Table 1:** The bioinformatic tools adopted for the prediction of secretory proteins from *Puccinia helianthi* transcriptome

Prediction algorithms	Objects predicted	References
SignalP v4.1	N-terminal signal peptides	http://www.cbs.dtu.dk/services/SignalP/
TMHMM v2.0	Transmembrane domains	http://www.cbs.dtu.dk/services/TMHMM/
Big-PI predictor	GPI-anchor site	http://mendel.imp.ac.at/gpi/fungi_server.html
TargetP v1.1	Secretion pathway and position	http://www.cbs.dtu.dk/services/TargetP/
ProtComp v9.0	Localization sequences	http://linux1.softberry.com/
LipoP v1.0	Lipoprotein signal peptides	http://www.cbs.dtu.dk/services/LipoP/
TatP v1.0	Signal peptide with RR-motif	http://www.cbs.dtu.dk/services/TatP/
Clustal Omega	Proteins homology prediction	http://www.ebi.ac.uk/Tools/msa/clustalo/

### Analysis of signal peptide sequences

In order to further examine the length of signal peptide sequences, the secretory proteins obtained from the previous step were analyzed using custom Perl script. Lipoprotein signal peptide prediction was done with LipoP v1.0, which was able to distinguish among lipoproteins (SPaseII-cleaved proteins), SPaseI-cleaved proteins, cytoplasmic proteins, and transmembrane proteins [41]. Signal peptides with an RR-motif were selected by TatP v1.0 and homology prediction of those signal peptide sequences was evaluated following alignment by Clustal Omega.

### ES proteins annotation

Predicted ES proteins were annotated with InterProScan and gene ontology (GO) terms for protein domain and family classification [42]. GO term enrichment analysis was performed using the DAVID bioinformatics resource [43]. KAAS (KEGG Automatic Annotation Server) performed functional annotation by BLAST search against the manually curated KEGG database [44] and provided insight into BRITE functional hierarchies and KEGG pathway maps [45]. The ES proteins were independently assessed for homology matches against NCBI’s non-redundant protein database and for orthologs against the Cluster of Orthologous Groups of proteins (COG) database using BLAST with permissive (E-value: 1e-10) search strategies. Finally, the ES proteins were predicted to have pathogenic function by BLAST analysis of the Pathogen Host Interaction (PHI) database (identity > 25, E-value: 1e-10).

## Results

### ES protein prediction from the transcriptome data set of *P. helianthi*

A total of 2,350 (6.7%) out of 35,286 ORFs were predicted as classical secretory proteins with SignalP. According to TMHMM v2.0 tool prediction, 149 (6.3%) proteins have two or more transmembrane helices, 422 (18.0%) proteins have one transmembrane helix, and 1,779 proteins lack transmembrane helices, accounting for 75.7% among 2350 proteins with N-terminal signal peptides. The remaining 1,779 proteins without transmembrane helices were queried with big-PI Predictor yielding 22 potential GPI-anchored proteins that may not be extracellularly secreted and 1,757 non GPI-anchored proteins ORFs.

TargetP v1.1 software was used to predict mitochondrial proteins. Among 1,757 proteins, 1,676 (95.4%) proteins had extracellular targeting signals, 68 (3.9%) proteins contained mitochondria targeting signals and 15 proteins (0.9%) contained other targeting signals.

The application of ProtComp v9.0 to the remaining 1,676 ORFs yielded a total of 908 ORFs (54.2%) as ES proteins (Additional file 2) and the remaining 768 proteins were predicted to be transported to the mitochondria (11.3%), cell membrane (14.9%), nucleus (3.8%), golgi (2.9%), cytoplasm (3.0%), endoplasmic reticulum (4.4%), lysosome (2.9%), peroxisome (1.3%) and vacuole (1.6%).

### ORF length of the secretory proteins from *P. helianthi*

**Fig. 1:**
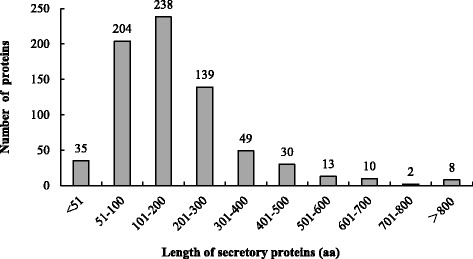
Length distribution of *Puccinia helianthi* ORFs coding secretory proteins

To examine the ORF length of the predicted secretory proteins from *P. helianthi*, 35,286 *P. helianthi* ORFs were analyzed by bioinformatics tools and 908 (2.6%) ORFs were identified as secretory proteins. Among them, 728 proteins contained the complete ORF. The longest protein was 1001 amino acids (aa) and the shortest one was 34 aa. The length of most secretory proteins (79.8% of the total identified proteins with a complete ORF) was between 51 and 300 aa. Within this group, 41.0% of them were 101–200 aa long. Thus, we suggest most secretory proteins probably fall in the shorter length range (Fig. 1).

### Characteristics of signal peptides of predicted secretory proteins in *P. helianthi*

**Fig. 2:**
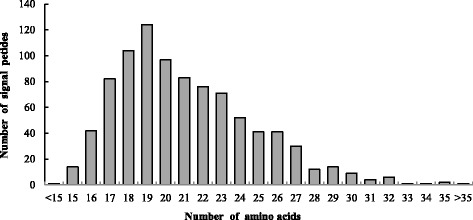
Length distribution of *Puccinia helianthi* signal peptides

The analysis of the signal peptides of 908 predicted secretory proteins reveals the length of the signal peptide ranges from 10 to 34 aa (mean = 21 aa) and most signal peptides (35.8%) ranged from 18 to 20 aa. Signal peptides with 19 aa length, however, were the most abundant, accounting for 13.7% (Fig. 2). The alignment of all 908 signal peptide sequences was done by Clustal Omega. The homology among the signal peptide sequences was low with the highest similarity (66.7%) observed between signal peptide sequence KU994941 and KU994981. No protein with an RR-motif signal peptide was found by TatP v1.0 while 463 proteins contained secretory pathway signal peptides cleavable by SpaseI, and 55 proteins harbored lipoprotein signal peptides cleavable by SpaseII. N-terminal transmembrane helices were found in 30 proteins and 360 of them could be localized to cytoplasmic organelles. Thus, most of the secretory proteins were determined to be secreted through the general secretion pathway (Sec-pathway).

### Amino acid composition of signal peptides of predicted secretory proteins in *P. helianthi*

**Fig. 3:**
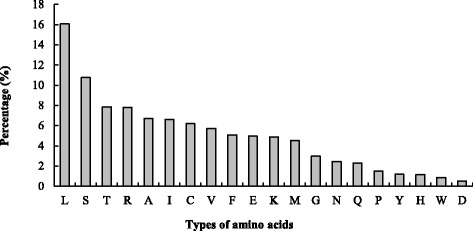
Percentage of 20 amino acid residues in *Puccinia helianthi* secretory protein signal peptides

The distribution of 20 amino acids in the signal peptide was statistically analyzed and the frequencies of amino acid residues in a descending order were: L - S - T - R - A - I - C - V - F - E - K - M - G - N - Q - P - Y - H - W - D. Hydrophobic amino acid leucine (L) showed an appearance rate of 16.1%, followed by serine (S) as 10.8% (Fig. 3). The occurrence of the negatively charged hydrophilic amino acid aspartate (D) is the lowest, accounting for 0.5%.

In general, the C-terminal region of signal peptides contains an enzyme recognition site. Based on this cleavage site, the amino acids of negative direction were named as −1, −2, and −3; those of positive direction were named as +1, +2, and +3. Between protein cutting locus positions −3 and +3, valine (V) is most likely to occupy the position −3 at a frequency of 26.7%. The frequency of serine (S) being at position −2 is 16.5%, alanine (A) has a 49.1% chance to be at position −1, while 12.9% of the time glutamine (Q) is found in position +1 (Table 2). Interestingly, it was found that most amino acids were widely used in the range of cleavage site −3 to +3 position in sunflower rust but no H, K, or Y was observed at position −1. This indicates amino acids near the cleavage site are highly polymorphic in sunflower rust.

**Table 2:** Amino acids frequency and distribution in cleavage sites of signal peptide of secretory proteins

Kinds of aa	20 amino acid residues at the cleavage position from - 3 to + 3 of the signal peptides											
	−3		−2		−1		+1		+2		+3	
	No.	Percentage (%)	No.	Percentage (%)	No.	Percentage (%)	No.	Percentage (%)	No.	Percentage (%)	No.	Percentage (%)
V	242	26.7	34	3.7	3	0.3	50	5.5	34	3.7	72	7.9
A	147	16.2	40	4.4	446	49.1	94	10.4	18	2.0	53	5.8
S	135	14.9	150	16.5	148	16.3	96	10.6	97	10.7	72	7.9
T	103	11.3	42	4.6	47	5.2	44	4.8	57	6.3	68	7.5
I	81	8.9	27	3.0	1	0.1	30	3.3	36	4.0	76	8.4
C	72	7.9	14	1.5	70	7.7	15	1.7	22	2.4	43	4.7
L	45	5.0	137	15.1	18	2.0	84	9.3	59	6.5	89	9.8
G	34	3.7	9	1.0	123	13.5	33	3.6	29	3.2	36	4.0
F	6	0.7	53	5.8	3	0.3	30	3.3	25	2.8	35	3.9
H	6	0.7	48	5.3	0	0.0	33	3.6	22	2.4	24	2.6
R	6	0.7	26	2.9	6	0.7	41	4.5	34	3.7	13	1.4
K	6	0.7	24	2.6	0	0.0	30	3.3	50	5.5	38	4.2
N	5	0.6	57	6.3	3	0.3	40	4.4	60	6.6	49	5.4
E	5	0.6	77	8.5	5	0.6	66	7.3	86	9.5	39	4.3
M	4	0.4	15	1.7	4	0.4	11	1.2	4	0.4	12	1.3
Y	4	0.4	31	3.4	0	0.0	26	2.9	18	2.0	16	1.8
Q	3	0.3	73	8.0	10	1.1	117	12.9	42	4.6	45	5.0
W	2	0.2	15	1.7	3	0.3	3	0.3	4	0.4	13	1.4
D	1	0.1	31	3.4	5	0.6	58	6.4	59	6.5	43	4.7
P	1	0.1	5	0.6	13	1.4	7	0.8	152	16.7	72	7.9

### Annotation of excretory/secretory (ES) of *P. helianthi*

**Fig. 4:**
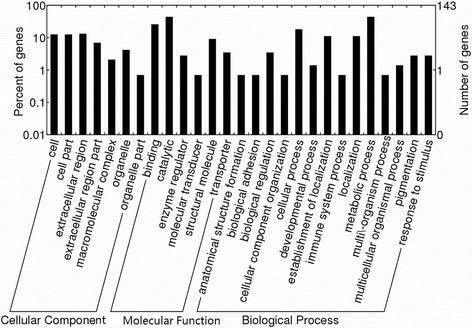
Gene ontology annotation of the secretory proteins of *Puccinia helianthi.* The best hits were aligned to the GO database, and 143 putative secretory proteins were assigned to at least one GO term. Most consensus sequences were grouped into three major functional categories and 27 sub-categories

All ES proteins identified were searched for sequence homology against our non-redundant dataset using BLAST. It was found that 581 (64.0%) computationally predicted ES proteins shared similarities with known proteins. A total of 143 ES proteins could be annotated in Gene Ontology (GO) and were classified into 27 functional groups, including 14 groups in biological process, seven in cellular component, and six in molecular function (Fig. 4). Within biological process, “metabolic process” (GO: 0008152) with 63 ES proteins and “cellular process” (GO: 0009987) with 26 ES proteins were predominant. In the category of cellular component, the three main groups were “extracellular region” (GO: 0005576, 19 ES proteins), “cell” (GO: 0005623, 18 ES proteins), and “cell part” (GO: 0044464, 18 ES proteins). The categories “catalytic activity” (GO: 0003824) and “binding” (GO: 0005488) were most common in molecular function, represented by 63 and 37 ES proteins, respectively.

ES proteins were subjected to GO enrichment analysis. The 10 top significant enriched GO terms are shown in Table 3. The hydrolase activity, hydrolyzing O-glycosyl compounds (GO:0004553), hydrolase activity (GO:0016787), hydrolase activity, acting on glycosyl bonds (GO:0016798), carbohydrate metabolic process (GO:0005975), peptidase activity, acting on L-amino acid peptides (GO:0070011), extracellular region (GO:0005576), peptidase activity (GO:0008233), serine-type endopeptidase activity (GO:0004252), serine-type peptidase activity (GO:0008236) and serine hydrolase activity (GO:0017171) are significantly enriched. These proteins included glycoside hydrolase, glucoamylase, phosphatase, phosphoesterase, lipase, cysteine peptidase, peptidase, cysteine-rich secretory protein, etc. Pathway associations were established for 82 (9.0%) ES proteins with the majority belonging to metabolism. The predicted ES protein dataset is comprised of important biological molecules, including enzymes, the spliceosome and the ribosome (Table 4).

**Table 3:** The 10 top GO terms significantly enriched for secretory proteins

GO term	GO-ID	Category	% of input genes in GO-term	*P*-Value
Hydrolase activity, hydrolyzing O-glycosyl compounds	GO:0004553	Molecular function	14.9	3.41E-20
Hydrolase activity	GO:0016787	Molecular function	3.7	7.09E-20
Hydrolase activity, acting on glycosyl bonds	GO:0016798	Molecular function	14.0	1.68E-19
Carbohydrate metabolic process	GO:0005975	Biological process	7.4	1.65E-16
Peptidase activity, acting on L-amino acid peptides	GO:0070011	Molecular function	7.1	5.09E-12
Extracellular region	GO:0005576	Cellular component	17.6	6.93E-12
Peptidase activity	GO:0008233	Molecular function	6.6	2.31E-11
Serine-type endopeptidase activity	GO:0004252	Molecular function	16.9	7.50E-11
Serine-type peptidase activity	GO:0008236	Molecular function	12.7	4.59E-10
Serine hydrolase activity	GO:0017171	Molecular function	12.7	4.59E-10

GO enrichment analysis was carried out using the hypergeometric test with a value threshold of 0.05. Most significantly enriched terms were selected according to their *p*-value

**Table 4:** Pathway categorization of the secretory proteins from *Puccinia helianthi*

Parent KEGG pathway	No. of ESPs	KEGG pathway in the category
Metabolism:	21	
Amino Acid Metabolism	1	Arginine and proline metabolism
Biosynthesis of Other Secondary Metabolites	2	Phenylpropanoid biosynthesis
Carbohydrate Metabolism	3	Galactose metabolism
	1	Propanoate metabolism
	4	Starch and sucrose metabolism
Energy Metabolism	1	Oxidative phosphorylation
Enzyme Families	3	Peptidases
Lipid Metabolism	2	Steroid biosynthesis
	1	Sphingolipid metabolism
Metabolism of Cofactors and Vitamins	2	Riboflavin metabolism
	1	Porphyrin and chlorophyll metabolism
Genetic Information Processing	17	
Folding, Sorting and Degradation	3	Chaperones and folding catalysts
	2	Protein processing in endoplasmic reticulum
	1	Ubiquitin system
	1	Ubiquitin mediated proteolysis
Replication and Repair	1	Base excision repair
	1	Chromosome
Transcription	4	Spliceosome
	1	Transcription factors
Translation	3	Ribosome Biogenesis
Environmental Information Processing	7	
Signal Transduction	1	Jak-STAT signaling pathway
	1	MAPK signaling pathway - yeast
	1	mTOR signaling pathway
	1	Notch signaling pathway
Signaling Molecules and Interaction	1	CAM ligands
	1	Cytokines
	1	Neuroactive ligand-receptor interaction
Cellular Processes	26	
Cell Communication	4	Focal adhesion
	1	Adherens junction
Transport and Catabolism	20	Lysosome
	1	Phagosome
Organismal Systems	7	
Digestive System	1	Salivary secretion
Endocrine System	2	PPAR signaling pathway
Immune System	2	Antigen processing and presentation
	2	Complement and coagulation
Human Diseases	4	
Infectious Diseases	1	African trypanosomiasis
	1	Tuberculosis
	1	Staphylococcus aureus infection
Neurodegenerative Diseases	1	Alzheimer’s disease

### Function prediction of predicted secretory proteins *in P. helianthi*

**Fig. 5:**
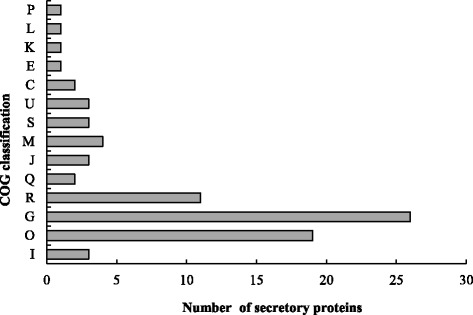
COG classifications of predicted secretory proteins in the transcriptome of *Puccinia helianthi.* All 80 putative proteins showing significant homology to those in the COG database were functionally classified into 14 families. Note: P, Inorganic ion transport and metabolism; L, Replication, recombination and repair; K, Transcription; E, Amino acid transport and metabolism; C, Energy production and transformation; U, Intracellular trafficking, secretion, and vesicular transport; S, Function unknown; M, Biosynthesis of cell and outer membrane; J, Translation, ribosomal structure and biogenesis; Q, The biosynthesis of secondary metabolites, transport and catabolism; R, General function prediction; G, The transport and metabolism of carbohydrates; O, Post-translational modification, protein turnover and molecular chaperones; I, Lipoid metabolism

Out of 908 secretory proteins queried against our non-redundant dataset using BLAST, 581 had functional descriptions, of which 279 had clear functional descriptions and 302 were predicted as hypothetical, conserved hypothetical, uncharacterized, or unnamed proteins. The querying of 908 secretory proteins against the COG database was performed for functional classification (Fig. 5). A total of 80 proteins could be assigned to the COG classification, of which 26 (32.5%) potentially participated in the transport and metabolism of carbohydrates (G; Fig. 5), followed by 23.8% involved in post-translational modifications, protein turnover, and molecular chaperones (O; Fig. 5). Proteins participating in inorganic ion transport and metabolism; replication, recombination and repair; transcription; amino acid transport and metabolism accounted for only 1.3%, respectively (P, L, K, E; Fig. 5). 188 out of the 908 proteins had annotations based on InterPro, of which 62 (33.0%) were hydrolases, including 19 peptidases, 15 glycoside hydrolases, seven esterases, five phosphatases, four each ribonuleases, and polysaccharide deacetylases, three each alpha/beta hydrolases, and glucanases (Table 5).

**Table 5:** Hydrolases among predicted secreted proteins of *Puccinia helianthi*

Classification	Gene code	Blastx InterPro
Peptidase	KU994901	Cysteine peptidase
	KU994902	Peptidase S1
	KU994903	Peptidase S8/S53 domain
	KU994904	Peptidase S8/S53 domain
	KU994905	Peptidase S8/S53 domain
	KU994906	Gamma-glutamyl transpeptidase
	KU994907	Peptidase S1
	KU994908	Peptidase S1
	KU994909	Cysteine peptidase
	KU994910	Peptidase C1A
	KU994942	Peptidase S1
	KU994952	Peptidase M36
	KU994953	Peptidase S10
	KU994954	Cysteine peptidase
	KU994955	Peptidase S1
	KU994982	Peptidase M28
	KU994983	Peptidase S1
	KU994984	Peptidase S8/S53 domain
	KU994985	Cysteine peptidase
Glycoside hydrolase	KU994943	Glycoside hydrolase
	KU994944	Glycoside hydrolase, family 31
	KU994945	Glycoside hydrolase, family 5
	KU994946	Glycoside hydrolase, family 31
	KU994947	Glycoside hydrolase, family 47
	KU994948	Glycoside hydrolase, family 10
	KU994949	Glycoside hydrolase, family 18
	KU994950	Glycoside hydrolase, family 30
	KU994951	Glycoside hydrolase, family 22
	KU994956	Glycoside hydrolase, family 61
	KU994957	Glycoside hydrolase, family 28
	KU994958	Glycoside hydrolase, family 30
	KU994959	Glycoside hydrolase
	KU994960	Glycoside hydrolase, family 65
	KU994971	Glycoside hydrolase, family 32
Esterase	KU994921	Cholinesterase
	KU994975	Cholinesterase
	KU994976	Palmitoyl protein thioesterase
	KU994977	Pectinesterase, catalytic
	KU994978	Palmitoyl protein thioesterase
	KU994979	Carboxylesterase, type B
	KU994980	Calcineurin-like phosphoesterase domain
Phosphatase	KU994928	Histidine phosphatase superfamily
	KU994929	Inorganic pyrophosphatase
	KU994930	Survival protein SurE-like Phosphatase/nucleotidase
	KU994931	Histidine phosphatase superfamily
	KU994932	Protein-tyrosine phosphatase
Nuclease	KU994922	Ribonuclease H-like domain
	KU994923	Deoxyribonuclease II
	KU994924	Ribonuclease H-like domain
	KU994925	Ribonuclease T2-like
Polysaccharide deacetylase	KU994986	Polysaccharide deacetylase
	KU994987	Polysaccharide deacetylase
	KU994988	Polysaccharide deacetylase
	KU994989	Polysaccharide deacetylase
Alpha/beta hydrolase	KU994972	Alpha/beta hydrolase fold-1
	KU994973	Alpha/Beta hydrolase fold
	KU994974	Alpha/beta hydrolase fold-1
Glucanase	KU994933	Glucanases superfamily
	KU994934	Glucanases superfamily
Glucoamylase	KU994926	Glucoamylase
Ceramidase	KU994927	Neutral/alkaline nonlysosomal ceramidase
Lipase	KU994935	Lipase

Peptidase, glycoside hydrolase, pectinesterase, polysaccharide deacetylase, pectate lyase and glucanosyltransferase were found possibly to be related to cell wall degradation. Nine proteins contained an MD-2-related lipid-recognition (ML) domain, six contained a lipocalin/cytosolic fatty-acid binding domain, and three contained a tyrosinase copper-binding domain. Six were annotated as lipocalin, four as the proteinase inhibitor I25 cystatin, four as apolipoprotein, three each as ribosomal protein, one as thaumatin, and two were annotated as the cysteine-rich allergen V5/Tpx-1-related secretory protein. The functions of most predicted secretory proteins are still unknown.

Blasting PHI yielded a total of 43 secretory proteins that could be correlated to pathogenicity (Tables 6 and 7). Of these, three secretory proteins (KU994907, KU994919 and KU994955) were predicted to be similar to an effector (plant avirulence determinant, Phibase accesstion ID: PHI: 653, PHI: 653 and PHI: 652, respectively) of *P. sojae* (Table 7).

**Table 6:** Pathogen Host Interaction database classification of secretory proteins of *Puccinia helianthi*

Category	Num of PHI	Proportion (%)
Reduced virulence	21	48.84
Unaffected pathogenicity	13	30.23
Loss of pathogenicity	4	9.30
Effector (plant avirulence determinant)	3	6.98
Mixed outcome	2	4.65

**Table 7:** Functional classes of the secretory proteins of *Puccinia helianthi*

PHI Category	Gene Code	Blastx NCBI Nr	Blastx InterPro
	KU994910	Hypothetical protein	Peptidase S8/S53 domain
	KU994911	Hypothetical protein	
Reduced virulence	KU994912	Putative polysaccharide lyase family 1	
	KU994925	Hypothetical protein	Ribonuclease T2-like
	KU994936	L-ascorbate oxidase	Multicopper oxidase
	KU994937	Hypothetical protein	Glucanosyl transferase
	KU994938	Hypothetical protein	
	KU994939	Hypothetical protein	
	KU994940	Predicted protein	
	KU994945	Hypothetical protein	Glycoside hydrolase
	KU994949	Chitinase	Glycoside hydrolase
	KU994961	Hypothetical protein	
	KU994962	Hypothetical protein	Pectate lyase/Amb allergen
	KU994963	Aspartic protease	
	KU994964	Retrotransposable element	
	KU994965	PR1 protein precursor	Cysteine-rich secretory protein
	KU994966	Niemann-Pick C1 protein	
	KU994967	Hypothetical protein	
	KU994968	Pathogenesis-related protein 1	
	KU994969	Hypothetical protein	
	KU994970	Hypothetical protein	Thioredoxin
Unaffected pathogenicity	KU994905	Probable serine carboxypeptidase CPVL	Serine carboxypeptidase
	KU994913	Beta glucosidase precursor	
	KU994914	Putative chaperone protein	DnaJ domain
	KU994915	Aspartic peptidase A1	
	KU994916	Beta glucosidase precursor	
	KU994943	Hypothetical protein	Glycoside hydrolase
	KU994947	Hypothetical protein	Glycoside hydrolase
	KU994957	Predicted protein	Glycoside hydrolase
	KU994972	Gastric triacylglycerol lipase-like	Alpha/beta hydrolase fold-1
	KU994974	Hypothetical protein	Alpha/beta hydrolase fold-1
	KU994975	Esterase	Cholinesterase
	KU994976	Esterase 10	Cholinesterase
	KU994980	Esterase 9	Carboxylesterase
Loss of pathogenicity	KU994917	Copper/zinc superoxide dismutase	
	KU994918	Ras-like C3 botulinum toxin substrate 1	Small GTPase superfamily
	KU994944	Hypothetical protein	Glycoside hydrolase
	KU994946	Hypothetical protein	Glycoside hydrolase
Effector	KU994907	Limulus factor D	Peptidase S1
	KU994919	Ovochymase-1	
	KU994955	Transmembrane protease serine 9-like	Peptidase S1
Mixed outcome	KU994920	Guanine nucleotide-binding protein	WD40 repeat
	KU994929	Inorganic pyrophosphatase	Inorganic pyrophosphatase

## Discussion

Protein is the major functional component of living organisms. Many pathogenic microbes can secrete proteins into host cells to promote their infection process [46]. Therefore, analysis of secretory proteins in the pathogen genome or transcriptome will help reveal pathogenic mechanisms. According to the signal peptide hypothesis [47], secretory protein destination is determined by its signal peptide. The signal peptide will be cleaved off when the protein reaches its destination. A free online program, SignalP, has been developed that accurately identifies eukaryotic signal peptides [48, 49]. An analysis of 47 known secretory protein and 47 other proteins of *C. albicans* by SignalP v2.0 showed that the putative results obtained were credible [30].

Signal peptides structures from various proteins commonly contain a positively charged N-region, a hydrophobic H-region and a neutral polar C-region. In the C-terminal region, helix breaking proline and glycine residues and small uncharged residues which are often found at the positions −3 and −1 determine the signal peptide cleavage site [50]. In *P. helianthi,* valine was observed more frequently (26.7%) at position −3, alanine was most likely to be at position −1 (49.1%), while histidine, lysine, tyrosine were not observed at this position. This indicates amino acids at −3 and −1 positions are relatively conserved, which might guarantee the recognition accuracy of signal peptidases.

Numerous algorithms are freely available for the prediction of protein structures, functions and interactions. Analyses of entire *S. cerevisiae* genome databases have included identification of GPI-anchored proteins [51], a prediction of protein sub-cellular localization [52] and a prediction of the “typical” secretory protein with Internet-based software SignalP v3.0, TargetP v1.01, Big-PI predictor and TMHMM v2.0 [33]. Bioinformatics approaches made the large scale prediction and analysis of ES proteins of Helminths possible, which included a comprehensive BLAST analysis to annotate the function of the ES proteins [53]. Thus, one approach to rapidly analyze the entire *P. helianthi* transcriptome and to predict its secretome is to utilize a wide range of appropriate and efficient bioinformatics tools.

After screening 35,286 ORFs of transcriptome data, 908 (2.6%) were predicted as secretory proteins*.* These putative secretory proteins were small proteins. Up to 79.8% of these secretory proteins were between 51 and 300 aa with signal peptide length between 18 and 20 aa. The short length of amino acids in secretory proteins is likely due to the reference genome of *P. helianthi* is not available and the unavoidable limitations of *de novo* transcriptome reconstruction. In signal peptides, the frequency of leucine (L), a hydrophobic amino acid, reached 16.1%. Abundant hydrophobic amino acids may be relevant to the secretion of secretory proteins and their subsequent destination. Most of the amino acids in signal peptides were aliphatic, which are mostly neutral amino acids or hydroxyl or sulfur amino containing amino acids. These amino acids may be important for physiochemical properties of the secretory proteins, which can make the signal peptide cross the plasma membrane easier and enhance signal guidance function. Prediction result showed most of the signal peptides of 908 putative secretory proteins were cleaved by SpI. The majority of the secretory proteins in *P. helianthi* are likely transported via the general secretory pathway. Furthermore, no signal peptide contained the RR-motif, which may indicate the Tat pathway does not exist or has minor roles in *P. helianthi*.

Signal peptides can guide the secretory proteins to subcellular locations, and play a key role in the process of metabolism. Signal peptide sequence analysis of all 908 secretory proteins showed sequence similarity is low, which indicates higher sequence variability, consistent with previous reports [34]. The low conservation might contribute to accurate positioning and specific metabolic functions of individual secretory proteins.

Among the 908 secretory proteins, most with functional descriptions are proteins responsible for transport and metabolism of carbohydrates, which is similar to previous research on *Bradyrhizobium japonicum* [54] and *Rhizobium etli* [55]. This implies a great deal of materials needed for rust pathogen development and infection may involve sugars, inorganic salt, and organic small molecules, which can be used as cofactors and to meet pathogen energy requirements. Our GO enrichment analysis indicated that hydrolase activity, carbohydrate metabolic process, peptidase activity were significantly enriched in the putative secretory proteins. It suggests rust pathogen *P. helianthi* can secrete various types of extracellular hydrolases which may include nucleases that can degrade the genetic material of the host plants and interfere with the host genetic metabolism. Additional hydrolase enzymes may be responsible for cell wall degradation; thereby making the host conducive to rust pathogen colonization by destroying the host cell structure and accelerating the process of infection. In addition, the secretory proteins also contain relatively unique serine proteases and similar proteins. In fungi, serine proteases are closely linked with pathogen infection and are often used to degrade the host plant proteins [56]. This suggests serine proteases may also be associated with the rust infection process. Cysteine peptidases (CPs) play important roles in facilitating the survival and growth of mammalian parasites [57]. CPs found in the sunflower rust pathogen, in turn, could also be associated with virulence to the host. In addition, two cysteine-rich secretory proteins identified as calcium chelating serine proteases [58] could be candidate effectors of this pathogen [59]. Three proteins similar to effectors of *P. sojae* were also found that might be similarly correlated with the pathogenicity of *P. helianthi*. These candidate proteins may provide more insight into common pathogenesis pathways utilized by both *P. sojae* and *P. helianthi* but more experimental evidence is necessary to confirm the biological roles of *P. helianthi* effectors.

Proteins containing the conserved ML domain are involved in lipid recognition or metabolism and are particularly important for the recognition of pathogen-related processes such as lipopolysaccharide (LPS) binding and signaling [60]. LPS and glycoproteins have been detected in the neck region of haustoria [61]. Proteins containing the ML domain in *P. helianthi* may, therefore, play a role in the recognition of host lipid-related products.

The thaumatin protein is considered a model pathogen-response protein domain for pathogenesis-related (PR) proteins involved in systematically acquired resistance and stress responses in plants, although their precise role is unknown [62]. Thaumatin-like secreted proteins of rust fungi may alter the plant-signalling pathway and have also been reported in the *Melampsora* secretome [63]. Future research into the role of thaumatin in sunflower rust infection will provide a better understanding of general and specific mechanisms of thaumatin-mediated resistance and pathogenesis.

Among these 908 secretory proteins in *P. helianthi*, the majority of them were unclassified due to rust fungi are biotrophic species and require specific genes in their life. The similar results were reported in wheat rust fungus *P. striiformis* f. sp. *tritici* [64, 65].

## Conclusion

In this study, various open source bioinformatics tools were used to predict and analyze ES proteins from *P. helianthi* transcriptome. Out of 35,286 ORFs of transcriptome data, 908 (2.6%) were predicted as secretory proteins and most were short proteins. A BLAST analysis was used to annotate the function of the ES proteins and provided further evidence for some proteins as candidates participating in the infection process of *P. helianthi.* Blasting PHI yielded a total of 43 secretory proteins that could be involved in pathogenicity and three secretory proteins were predicted to be similar to the effectors of *P. sojae*. Therefore, this investigation provides a novel approach for identifying elicitors and pathogenic factors. It also establishes a sound foundation for understanding the structures and functions of the pathogenic factors of *P. helianthi.* In conclusion, our data can be used as a candidate gene resource for further computational or wet lab research to unveil the molecular mechanisms underlying the interaction between sunflower and *P. helianthi.*

## Additional files

Additional file 1: Dataset of 35286 ORFs of the *Puccinia helianthi* transcriptome (XLSX 4749 kb)
Additional file 2: Dataset of 908 putative secretory proteins (XLSX 129 kb)

## Abbreviations

aa: Amino acid
COG: Cluster of Orthologous Groups of proteins
CPs: Cysteine peptidases
CWDEs: Cell wall degrading enzymes
ES: Excretory/secretory
GO: Gene ontology
KAAS: KEGG Automatic Annotation Server
LPS: Lipopolysaccharide
Lsp: Lipoprotein-specific SPase
ML: MD-2-related lipid-recognition
PHI: Pathogen host interaction
PR: Pathogenesis-related
RR-motif: Twin-arginine motif
Sec-pathway: Secretion pathway
SpI: Signal peptidase I
SpII: Signal peptidase II
Tat: Twin-arginine translocation
